# miR194 hypomethylation regulates coronary artery disease pathogenesis

**DOI:** 10.1186/s12920-022-01421-7

**Published:** 2022-12-18

**Authors:** Lian Duan, Yongmei Liu, Jun Li, Yun Zhang, Jiangquan Liao, Yan Dong, Wang Jie

**Affiliations:** 1 Department of Cardiology, Guang Anmen Hospital, No. 5 Beixiange, Xicheng District, Beijing, China; 2 Department of Cardiology, China-Japan Friendship Hospital, No. 2 Yinghuayuan East Street, Chaoyang District, Beijing, China

**Keywords:** Coronary artery disease, DNA methylation, miRNA, *MAPK*, Apoptosis

## Abstract

Coronary artery disease (CAD) is one of the most common heart diseases, characterized by the hardening and narrowing of arteries, resisting blood supply to cardiac muscle. Despite extensive research, the pathogenesis and therapeutic options for CAD remain limited. Epigenetic regulation plays a critical role in CAD progression. Here, we report a unique DNA methylation-miRNA-mRNA regulatory network for CAD, delineated through DNA methylation assays, miRNA and mRNA sequencing, bioinformatics analyses. We also identified key signaling pathways in this network, including the *miR194* promoter-*miR194*-*MAPK* signaling pathway by pyrosequencing, methylation PCR, qRT-PCR. This pathway could play a role in CAD by apoptosis. Our findings suggested that this signaling pathway may be a potential therapeutic target for CAD. We believe that our study significantly contributes to an improved understanding of the role of specific miRNAs methylation, miRNA, and mRNAs in CAD pathogenesis.

**Supplementary Information:**

The online version contains supplementary material available at 10.1186/s12920-022-01421-7.

## Introduction

Coronary artery disease (CAD) is one of the most common types of heart disease characterized by the hardening and narrowing of arteries, resisting blood supply to cardiac muscle. Despite extensive research, the pathogenesis and therapeutic options for CAD remain limited. Interestingly, recent research has highlighted the role of epigenetics in cardiovascular development and disease [1]. Epigenetic regulation includes DNA methylation, histone modifications, microRNAs (miRNAs), long non-coding RNAs, and circular RNAs [2]. Several studies have illustrated that miRNAs have great potential as cardiovascular biomarkers [3].

miRNAs are small endogenous non-coding RNA molecules composed of 19–22 nucleotides that affect various biological processes, such as development, differentiation, apoptosis, and cell proliferation, by regulating gene expression. Cardiac-specific miRNAs, including miR-1, miR-133, miR-208, and miR-30b, exhibit some critical diagnostic value [4–7]^.^ DNA methylation refers to the addition of a methyl group on the fifth carbon atom of 5ʹ- CpG (cytosine-phosphate-guanine)-3ʹ through the action of DNA methyltransferase (DNMT). DNA methylation is involved in various pathophysiological activities, such as time- and space-specific gene expression, X-chromosome inactivity, aging, cancer, and cardiovascular disease [8, 9]. It is stable and can be influenced by environmental and genetic factors. DNA methylation is often localized to the promoter region, regulating gene expression [10]. DNA hypermethylation on the promoter of the gene inhibits the gene expression, whereas hypomethylation activates it. A systematic review involving 32 DNA methylation studies on CAD [10] and a large-sample cohort with 13,356 individuals was conducted to ascertain the correlation of coronary heart disease events with DNA methylation [11]. The same effect of DNA methylation may be observed in miRNAs. *miR-223* can influence the development of atherosclerosis and ischemic stroke, with hypomethylation of the promoter being related to atherosclerotic cerebral infarctions [12]. Therefore, we hypothesized that the epigenetic regulation of DNA methylation and miRNAs occurs together in the development of CAD[13], and that they may form a regulatory network involving transcriptional and epigenetic control of gene expression.

Here, we report the epigenetic regulation of DNA methylation and miRNAs that occur in CAD and may form a regulatory network involving transcriptional and epigenetic genes.

## Results

### Data preprocessing and differentially methylated regions (DMRs) and differentially expressed genes (DEGs)

**Fig. 1:**
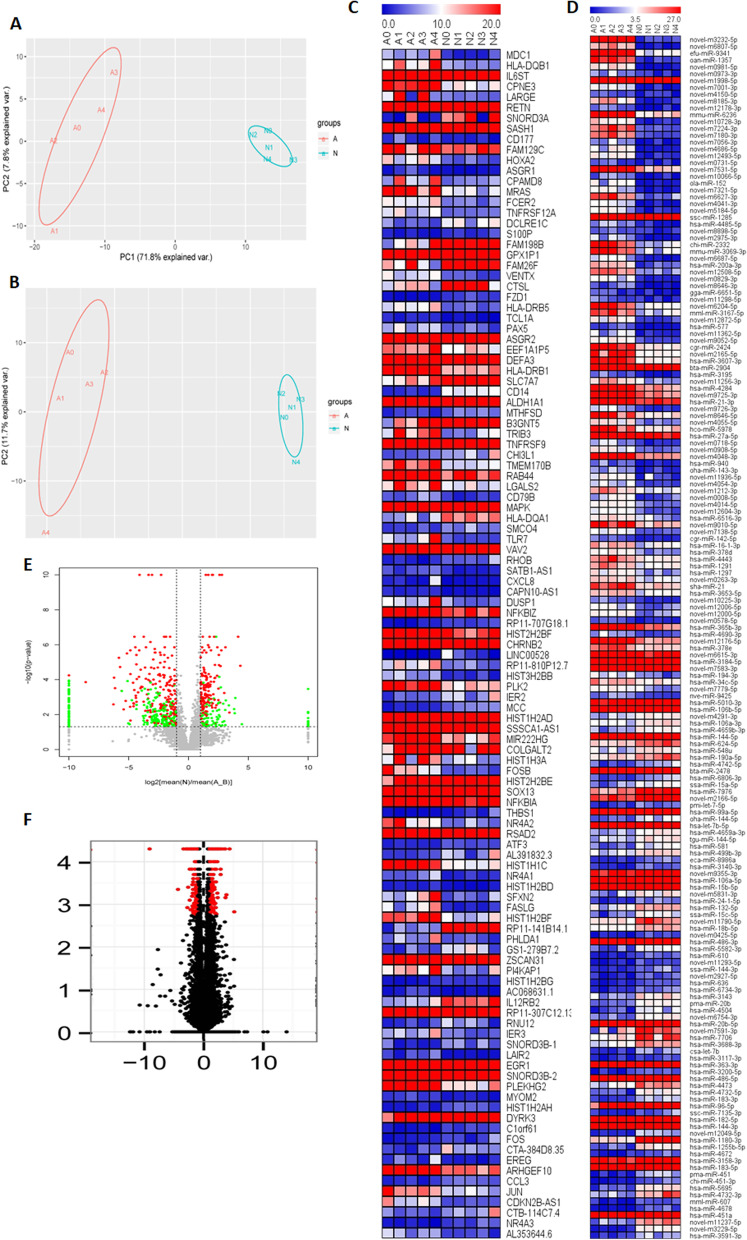
miRNAs and mRNAs are differentially expressed in CAD. **a** The PCA of DE miRNAs. **b** The PCA of DEGs. **c** The heatmap of DE miRNAs between CAD and control groups. (The first five samples are in CAD group such as A0-A4, and the last five samples in control group such as N0-N4. The key DE miRNAs and mRNAs were chosen to display with the criterion q < 0.005 and log2 > 1.5.) **d** The heatmap of DEGs between CAD and control groups. **e** The volcano plot of DE miRNAs. **f** The volcano plot of DEGs. The heatmap were created by the software MultiExperiment Viewer, version 4.6.0 (http://mev.tm4.org/)

We collected 88 blood samples from CAD patients (n = 53) and healthy controls (n = 35). First, we randomly chose 5 blood samples in two groups for DNA methylation-Seq, miRNA-Seq, and RNA-Seq. We obtained massive data of CpGs and transcripts in patient peripheral blood with CAD and healthy controls. Each gene expression profile revealed 8710 and 8580 known and novel miRNAs, respectively, and 32,300 mRNAs were detected. PCA also revealed distinct expression signatures of miRNA and mRNA between CAD and healthy control (Fig. 1a, b). Following analyses, we obtained 295 DE miRNAs, of which 171 and 124 were downregulated and upregulated, respectively (Additional file 1 and 2). We also acquired 470 DEGs, of which 220 and 250 were downregulated and upregulated, respectively. The respective DEG heat maps and volcano plots are presented in Fig. 1c, d, e, and f.

Following quality control, we detected 2.02 million CpGs in each sample. Using the screening criterion (coverage ≥ 5; 2. P value ≤ 0.05), we obtained a total of 28,461 DMRs, of which 4498 and 23,963 were hypermethylated and hypomethylated, respectively. When DMRs were matched to the differentially expressed miRNA promoter, 64 miRNAs and the corresponding promoter sites in the DMRs were selected. Besides, 203 DEGs in RNA-Sequencing matched the potential targets of 64 miRNAs. The information on the control and CAD groups in sequencing is shown in Table 1. There are differences in the two groups, such as hypertension, LDL, TG, aspirin administration, statins administration, Gensini scores.

**Table 1:** The information on the control and patients with CAD in sequencing

	control (n = 5)	CAD (n = 5)	P value
Age (years)	57.00 ± 8.15	66.4 ± 5.32	0.063
Gender (male%)	2 (40%)	3 (60%)	
Smoking history[n (%)]	1 (25%)	2 (40%)	
Hypertention [n (%)]	1 (20%)	4 (80%)	
Diabetes [n (%)]	2 (20%)	4(80%)	
TC (mmol/L)	2.75 ± 0.48	3.97 ± 0.82	0.021
TG (mmol/L)	1.26 ± 1.35	2.33 ± 1.47	0.263
LDL-C (mmol/L)	2.55 ± 0.51	3.07 ± 0.97	0.140
HDL-C (mmol/L)	1.39 ± 0.42	1.02 ± 0.16	0.096
Aspirin administration [n (%)]	0 (0%)	4 (80%)	
Statins administration [n (%)]	0 (0%)	3 (60%)	
Gensini scores	1.3 ± 1.1	59.6 ± 8.0	0.000

### Immune-related molecular functions and biological processes of DE RNAs play a role in CAD

**Fig. 2:**
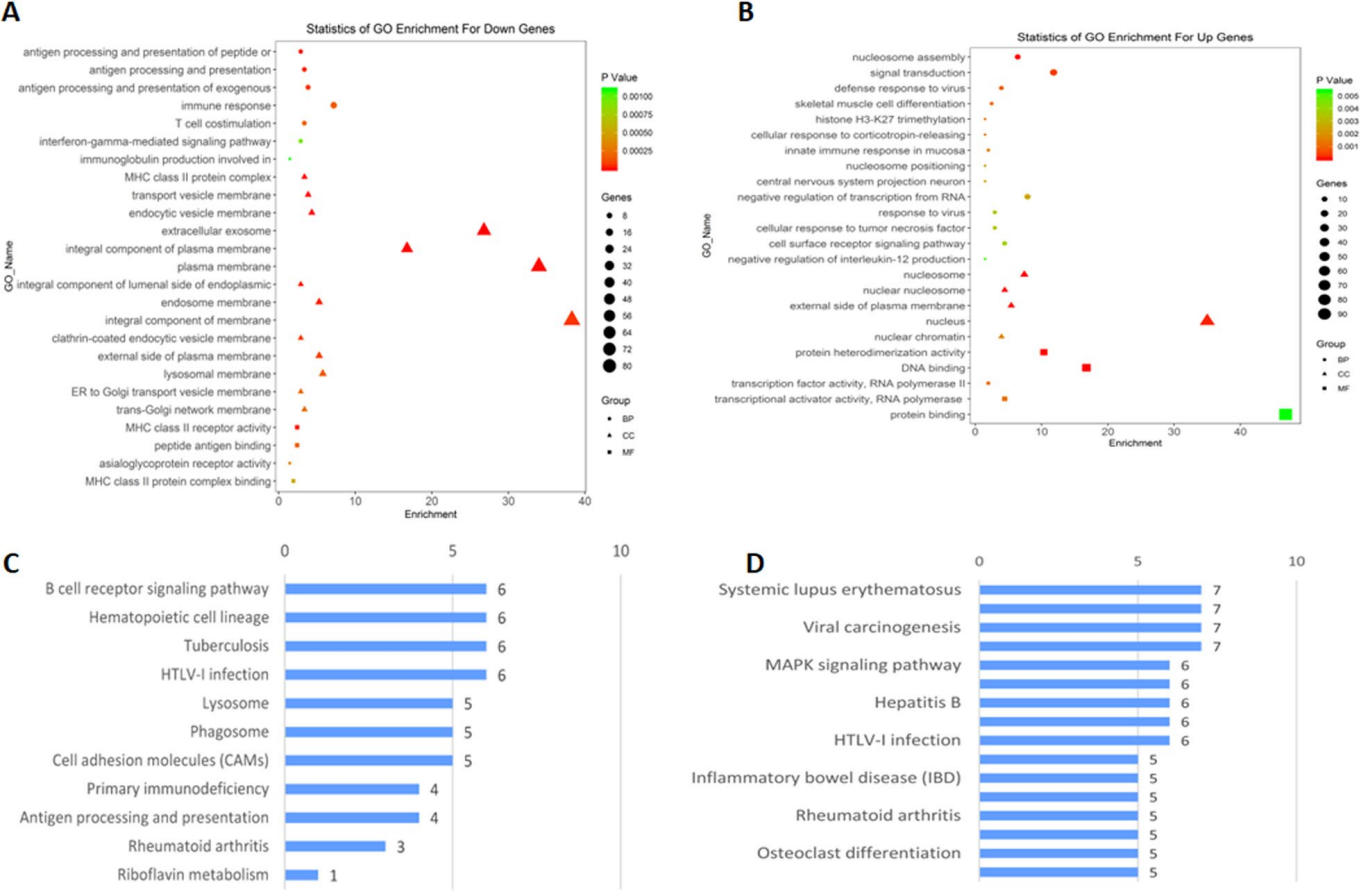
GO terms and KEGG pathways of DE mRNA in CAD. **a** The top 25 GO terms of downregulated genes. **b** The top 25 GO terms of upregulated genes. **c** The top KEGG pathways of downregulated genes. **d** The top KEGG pathways of upregulated genes

We identified CAD-related genes by performing functional and pathway enrichment analyses using DAVID 6.8 [14]. We identified the gene ontology (GO) functions of significantly different downregulated genes, 20 biological processes, 15 cellular components, and five molecular functions. The top 25 GO terms are shown in Fig. 2a. For upregulated genes, 27 biological processes, 5 cellular components, and 5 molecular functions were identified. The top 25 GO terms are shown in Fig. 2b. The top GO enrichment terms in downregulated mRNAs were associated with antigen processing and presentation, immune response, and T cell costimulation. The top GO enrichment terms in upregulated mRNAs were related to nucleosome assembly, signal transduction, defense response to a virus, histone H3-K27 trimethylation. Meanwhile, biological processes related to immunity and inflammation also play a role in GO enrichment, such as antigen processing and presentation, immune response, and T cell costimulation, and so on.

Through the KEGG (Kyoto Encyclopedia of Genes and Genomes) pathway enrichment [15–17], we obtained 26 and 19 pathways that had upregulated and downregulated mRNAs, respectively. The tops KEGG pathways in downregulated mRNAs comprised B cell receptor signaling pathway, hematopoietic cell lineage, tuberculosis, HTLV-I infection, lysosome, phagosome, cell adhesion molecules, primary immunodeficiency, antigen processing and presentation, rheumatoid arthritis. The tops KEGG pathways in upregulated mRNAs comprised systemic lupus erythematosus, viral carcinogenesis, mitogen-activated protein kinase (MAPK) signaling pathway, hepatitis B, HTLV-1 infection, inflammatory bowel disease, rheumatoid arthritis, osteoclast differentiation (Fig. 2c, d). The main pathways were related to immunity, such as the systemic lupus erythematosus, *MAPK* signaling, and B cell receptor signaling pathways.

### DMRs, DE miRNAs, and mRNAs formed gene–gene networks by mutual interaction

Methylation of miRNA promoters controls the miRNA production, influencing miRNA expression, consequently impacting the downstream mRNA expression. After obtaining the differentially expressed miRNA and mRNA, according to the regulatory relationship between genes, the UCSC database and Miranda miRNA target gene database was used (https://genome.ucsc.edu/), (https://omictools.com/miranda-tool/). The gene regulatory network was screened. If the DNA methylation miRNA and miRNA mRNA regulatory relationships obtained by the two methods are verified in the sequencing results, the gene pairs that are both potentially related and differentially expressed in the sequencing results are retained, that is, the gene pairs are differentially expressed and there is an intergenic regulatory relationship.

**Fig. 3:**
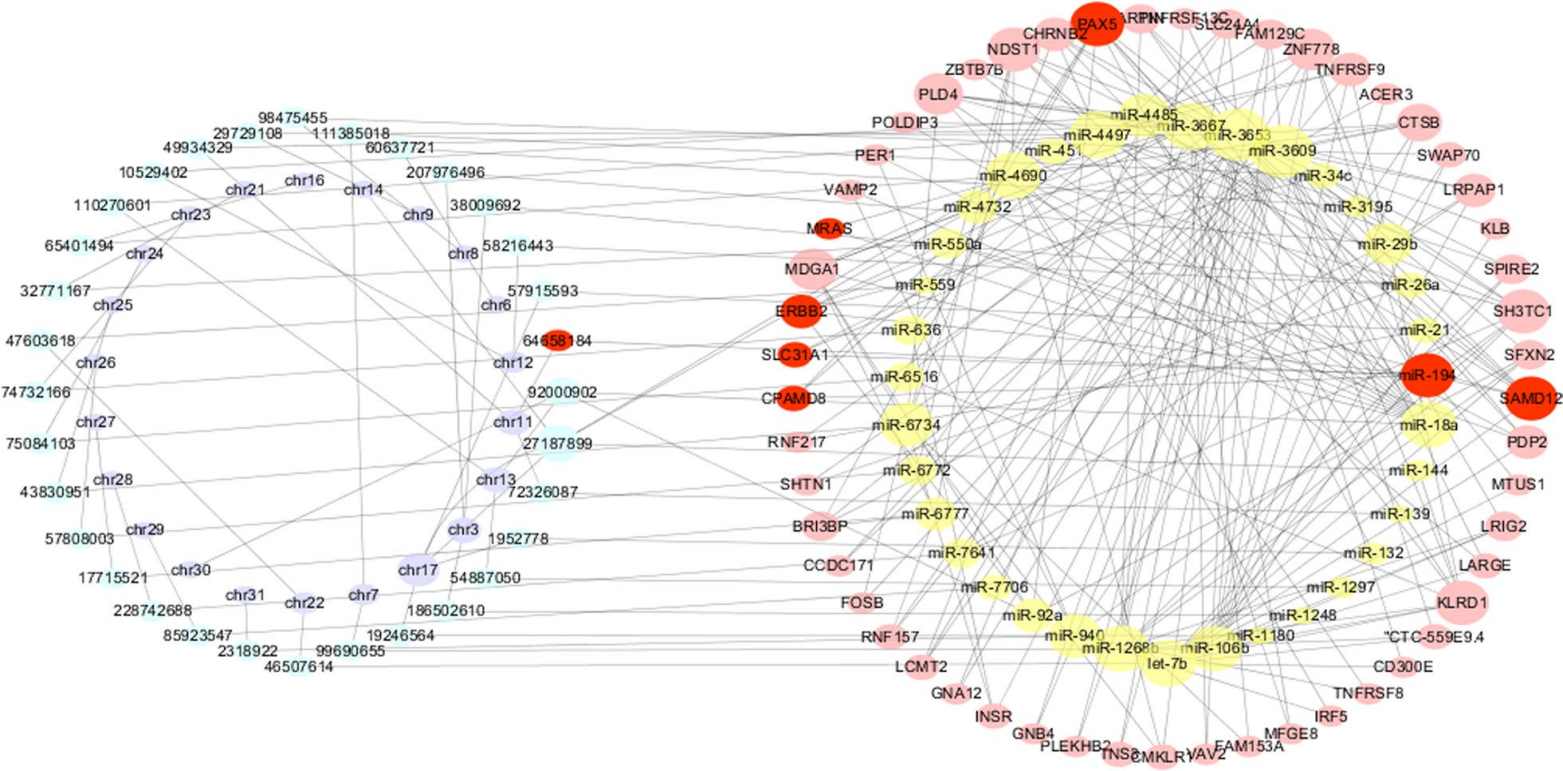
DMRs, DE miRNAs, and mRNAs formed gene–gene networks by mutual interaction. Purple circle is chromosome. blue circle is DMRs. Yellow circle is DE miRNA. Pink circle is DE mRNAs. Red circle is the gene related to miR194. Grey lines indicate the association of genes

We used the Pearson correlation coefficients and starBase to construct the gene–gene interaction network between DMR, miRNA, and mRNA. The network included 32 DMRs (five hypomethylated and 27 hypermethylated regions), 32 miRNAs (similar to DMR, 13 downregulated and 19 upregulated miRNAs), and 180 mRNAs (60 downregulated and 120 upregulated miRNAs; Fig. 3). We inferred that 28 DMRs-regulated miRNAs and that this subsequently changed the target expression.

### Hypomethylation of *miR194* regulates cell death and inflammation pathways

**Fig. 4:**
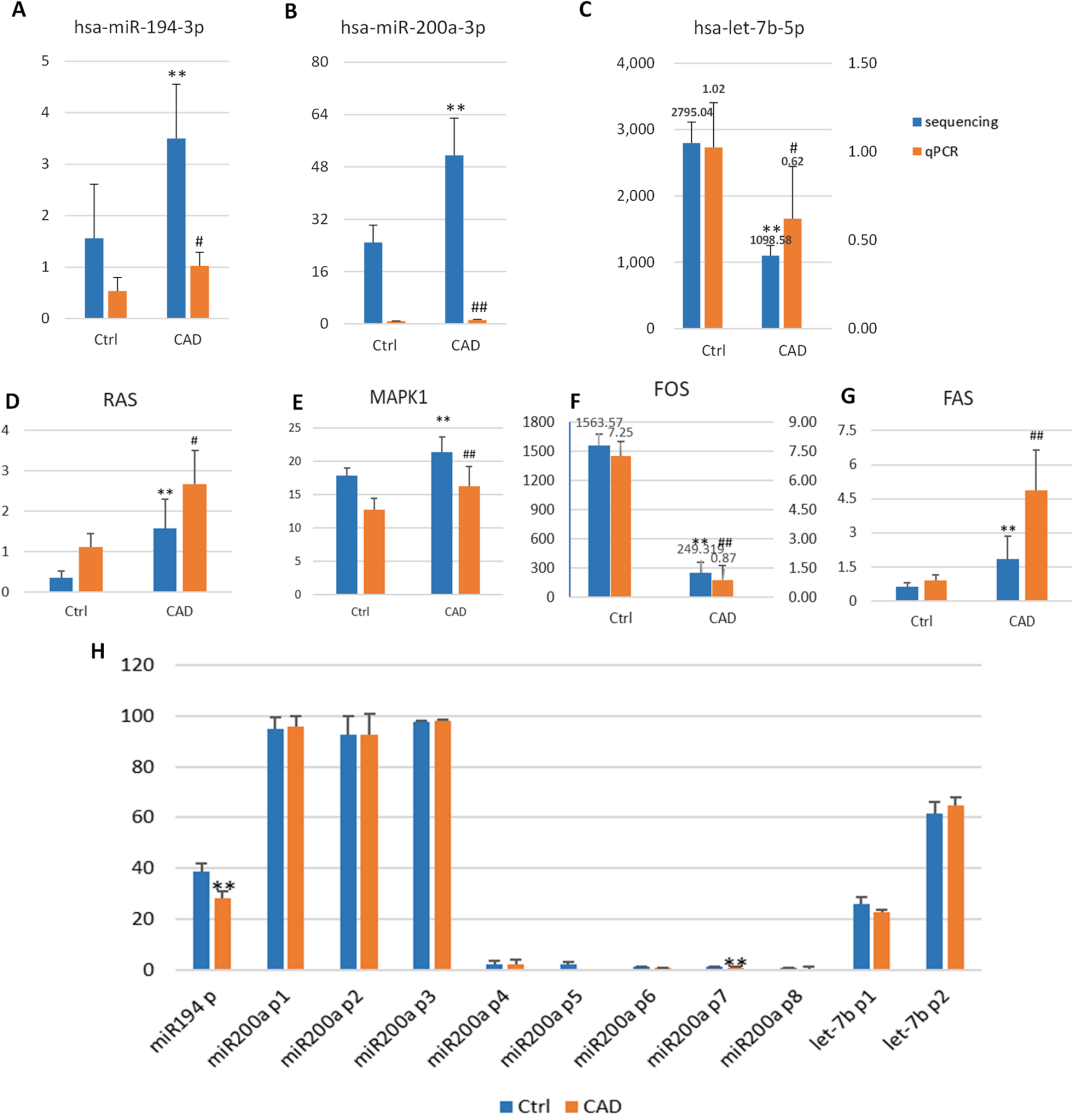
Relative expression levels of methylation, miRNA, and mRNA. (A-G) Orange column is qPCR data and blue sequencing data. **a** Sequencing and qPCR levels of *has-miR-194-3p*. **b** Sequencing and qPCR levels of *has-miR-200a-3p*. **c** Sequencing and qPCR levels of has-let-7b-5p. Because the expression level has too large gap, the left y coordinate axes is for sequencing data and the right for qPCR result. **d** Sequencing and qPCR levels of RAS. **e** Sequencing and qPCR levels of *MAPK1*. **f** Sequencing and qPCR levels of FOS. Because the expression level has too large gap, the left y coordinate axes is for sequencing data and the right for qPCR result. B Sequencing and qPCR levels of FAS. **h** Methylation levels of *miR-194*, miR-200a, let-7b with pyrosequencing. *P < 0.05, **P < 0.01, significantly different from the control group in sequencing. ^#^P < 0.05, ^##^P < 0.01, significantly different from the control group in qPCR

Three miRNAs were selected which has close relation with DMRs using formed gene–gene network. Using qRT-PCR, we detected *miR-194*, *miR-200a*, and *let-7b* in a validation cohort in Tab. 2 (n = 78, CAD 48 blood samples and healthy control 30 blood samples). All three miRNAs were differentially expressed in CAD. Similarly, we measured *RAS*, *MAPK1*, *FOS*, and *FAS* using qRT-PCR in the validation cohort. We found that *RAS*, *MAPK1*, *FOS*, and *FAS* were differentially expressed in CAD. The expression patterns of these three miRNAs and four mRNAs in the validation cohort were similar to those in the sequencing cohort (Fig. 4a-g).

**Table 2:** The information on the control and patients with CAD in qRT-PCR

	Control (n = 30)	CAD (n = 48)	P value
Age (years)	49.40 ± 4.20	62.63 ± 7.63	0.000
Gender (male%)	12 (40%)	33 (68.8%)	
Smoking history [n (%)]	12 (40%)	10 (20.8%)	
Hypertention [n (%)]	12 (40%)	30 (62.5%)	
Diabetes [n (%)]	2 (6.7%)	13(27.1%)	
TC (mmol/L)	4.94 ± 0.88	3.52 ± 0.87	0.000
TG (mmol/L)	1.07 ± 0.57	1.9 ± 1.25	0.058
LDL-C (mmol/L)	2.90 ± 0.70	2.58 ± 0.82	0.300
HDL-C (mmol/L)	1.66 ± 0.49	0.98 ± 0.15	0.000
Aspirin administration [n(%)]	0 (0%)	37 (77%)	
Statins administration [n(%)]	0 (0%)	35 (73%)	
Gensini scores	1.8 ± 2.86	47.17 ± 12.545	0.000

### Pyrosequencing verified hypomethylation of miR194

Three DMRs corresponding to unique miRNAs were verified using pyrosequencing, including miR-194-cg64658184, miR-200a-cg1095596, and let-7b-cg46507614. First, we analyzed the methylation of these three miRNAs in CAD and healthy controls. miR-194-cg64658184 and miR-200a-cg1095596 were hypomethylated and upregulated in CAD, whereas let-7b-cg46507614 was hypermethylated and downregulated. Thereafter, we conducted pyrosequencing to identify 11 CpGs from these three DMRs. We obtained two CpGs, miR-194 -cg64660076, and miR-200a -cg1167297 (P = 0.002, 0.044) between CAD and healthy controls (Fig. 4h).

### Methylation of *miR-194* and *miR-200a* in oxidative HUVECs with Sequenom MassARRAY

**Fig. 5:**
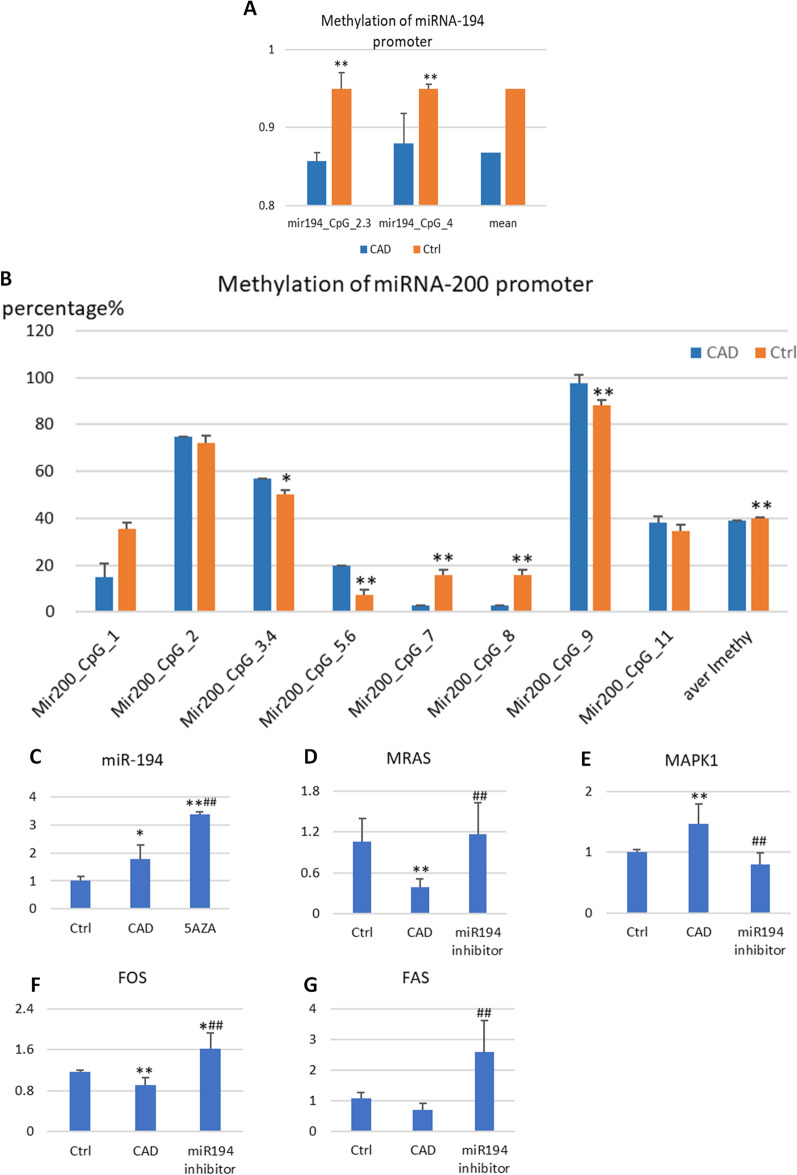
Methylation of miRNA-194 and miR-200a promoter regions and expressions of *miR-194*, FAS, MRAS, FASL, and *MAPK1*. **a** Methylation of miRNA-194 promoter regions. **b** Methylation of miR-200a promoter regions. *P < 0.05, **P < 0.01, significantly different from the CAD group. **c** miR194 **d** RAS **e** *MAPK1* **f** FOS **g** FAS. *P < 0.05, **P < 0.01, significantly different from the control group. ^#^P < 0.05, ^##^P < 0.01, significantly different from the CAD group

The Sequenom MassARRAY system was used to detect the methylation of *miR-194* and *miR-200a* in oxidative and normal HUVECs (Human Umbilical Vein Endothelial Cells). It was observed that *miR-194* promoters were hypomethylated in oxidative HUVECs in pyrosequencing, which matched the results of patients with CAD by sequencing (Fig. 5a). The methylation levels of *miR-200a* were inconsistent, whereas miR200a-CpG7, CpG8, and CpG9 were hypomethylated, and miR200a-CpG3.4 and CpG5.6 hypermethylated. Nevertheless, the mean of *miR-200a* methylation appeared hypomethylated in pyrosequencing as sequencing in CAD. (Fig. 5b).

### 5aza and *miR-194* inhibitor validated gene–gene interactions using qRT-PCR

In oxidative HUVECs, the level of *miR-194* increased with qRT-PCR (P < 0.05). 5-Aza is a DNA-hypomethylating agent. When HUVECs were hypomethylated, the expression of *miR-194* was enhanced (Fig. 5c). We then inhibited *miR-194* in HUVECs, the levels of *RAS*, *MAPK1*, and *FAS* were downregulated in oxidative HUVECs. When treated with *miR194* inhibitor, these three mRNAs were upregulated compared with oxidative HUVECs (P < 0.05; Fig. 5d-g).

### Pearson correlation between CpG island methylation and clinical characteristics of the study population

We performed the Pearson correlation analyses between miR194 promoter methylation, miR194 and clinical characteristics of the study population (n = 10) in Table 3. The result indicated miR194 promoter methylation was significantly associated with Gensini scores (r = − 0.772, p = 0.009), and miR194 was significantly related to LDL-C (r = − 0.716, p = 0.02), HDL-C (r = 0.667, p = 0.035), and Gensini scores (r = 0.834, p = 0.003).

**Table 3:** Pearson or Spearman correlation between miR194 promoter methylation and miR194

	Relationship coefficient (miR194 promoter methylation)	P value (miR194 promoter methylation)	RelationshipCoefficient (miR194)	P value (miR194)
Age	− 0.4	0.252	0.471	0.169
SBP	− 0.455	0.187	0.539	0.108
DBP	− 0.06	0.869	0.253	0.481
FPG	− 0.248	0.489	0.179	0.621
TC	− 0.438	0.205	0.626	0.053
TG	− 0.004	0.992	0.597	0.069
LDL-C	− 0.576	0.082	− 0.716	0.02
HDL-C	0.557	0.094	0.667	0.035
Gensini scores	− 0.772	0.009	0.834	0.003

Pearson correlation between miR194 promoter methylation and miR194. Data are summarized for binary variables

*SBP* systolic blood pressure, *DBP* diastolic blood pressure, *TC* total cholesterol, *HDL-C* high-density lipoprotein cholesterol, *LDL-C* low-density lipoprotein cholesterol, *TG* triglyceride, *FBG* fasting blood glucose

Pearson correlation between miR194 promoter methylation and miR194. Data are summarized for binary variables. SBP, systolic blood pressure; DBP, diastolic blood pressure; TC, total cholesterol; HDL-C, high-density lipoprotein cholesterol; LDL-C, low-density lipoprotein cholesterol; TG, triglyceride; FBG, fasting blood glucose.

### MiR194 inhibitor induction triggered apoptotic cell death, improved the cell cycle inhibition

**Fig. 6:**
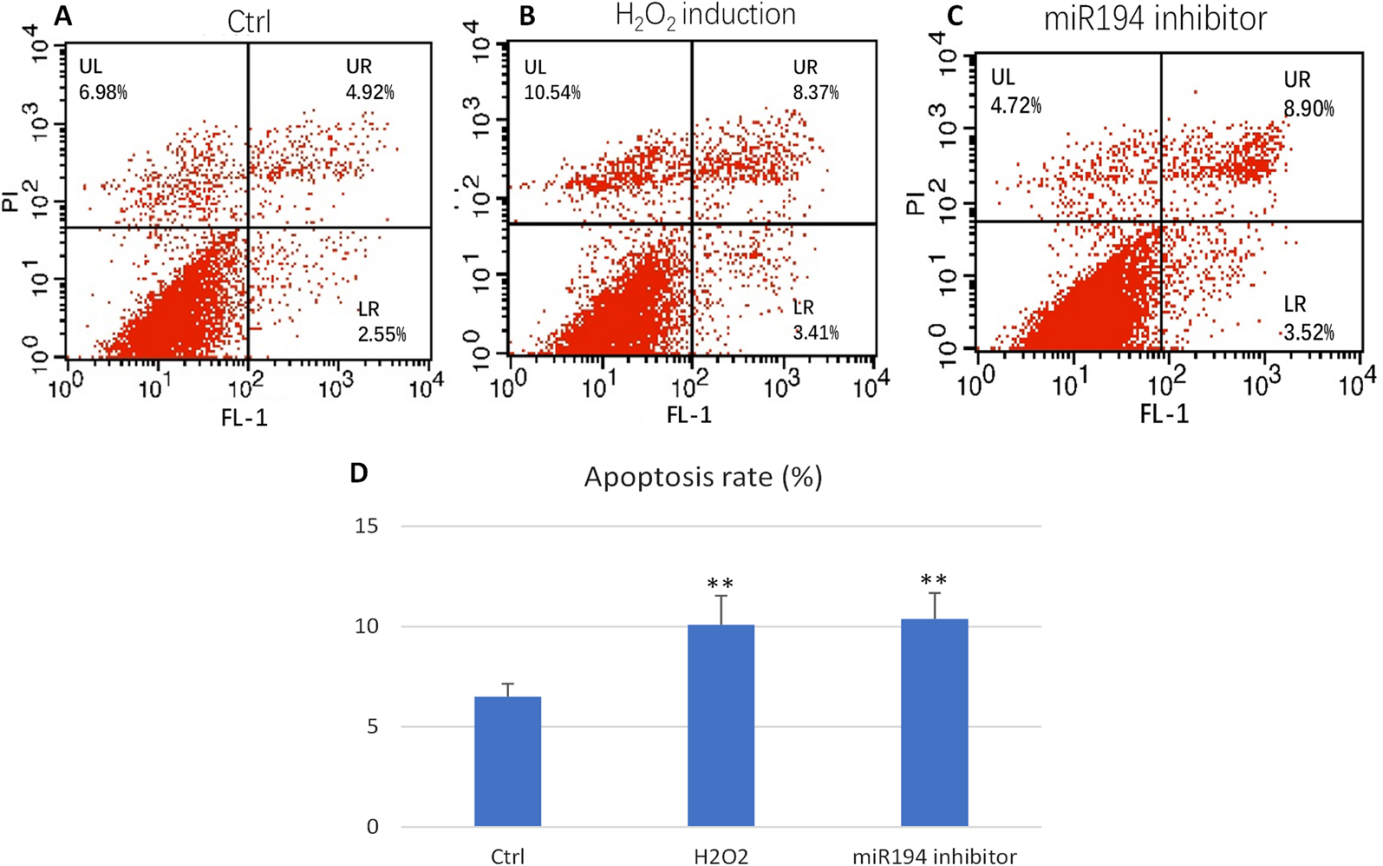
AnnexinV/PI apoptosis detection. **a** Control group. **b** H_2_O_2_ group. **c** miR194 inhibitor group **d** The level of apoptosis in HUVECs. **P < 0.01, significantly different from the control group

Using an AnnexinV/propidium iodide (PI) apoptosis assay, we detected the level of damaged early and late apoptotic and normal endothelial cells with oxidative damage (Fig. 6a-d), indicating that H_2_O_2_ treatment and *miR194* inhibitor induction triggered apoptotic cell death of HUVECs. However, miR194 inhibitor didn’t decrease apoptosis rate.

**Fig. 7:**
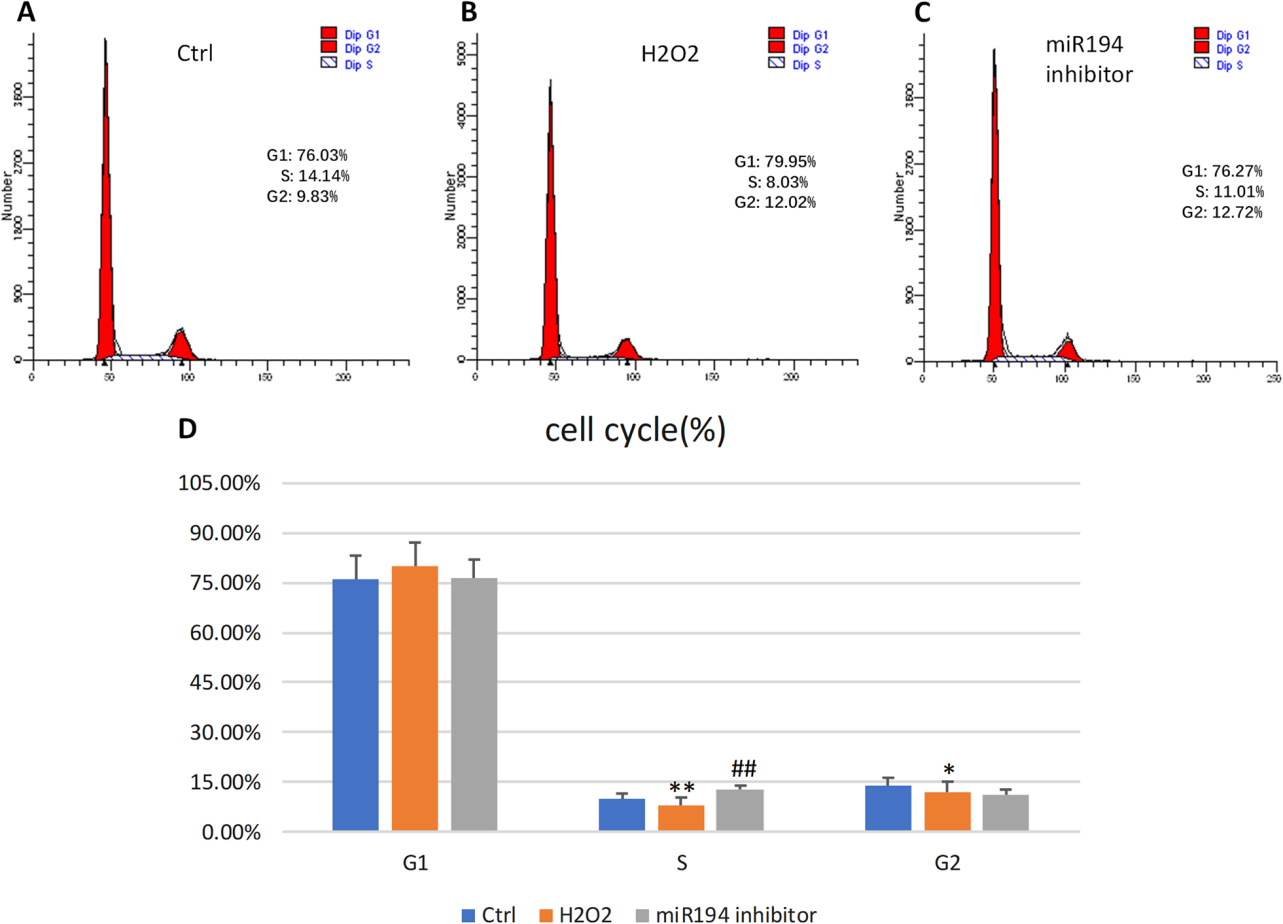
PI cell cycle detection. **a** Control group. **b** H_2_O_2_ group. **c** miR194 inhibitor group, **d** The cell cycle level in endothelial cells

Using PI cell cycle detection, H_2_O_2_ intervention can inhibit the proliferation of HUVECs. The S- and G2 phases of cell proliferation were reduced, with the S-phase at 8.03% and the G2 phase at 12.02% (Fig. 7a–d). The proportion of G0-G1 cells in the proliferation resting phase increased to 79.95%. Transfection with a *miR194* inhibitor improved the cell cycle inhibition, and the S-phase increased to 12.72%.

## Discussion

Despite extensive research on CAD-related methylation, DNA methylation and the associated miRNA-mRNA CAD regulation remain poorly understood. We screened 88 subjects for DNA methylation sites, miRNAs, and mRNAs in the CAD and normal control groups. High-throughput sequencing was used to randomly select five cases from each group to detect the level of DNA methylation, miRNA, and mRNA expression in peripheral blood mononuclear cells and to screen differential genes between the CAD and control groups. Correlation analysis was performed to construct a DNA methylation-miRNA-mRNA gene regulatory network. We screened and identified a total of 28,461, 295, and 470 differentially expressed methylation sites, miRNAs, and mRNAs, respectively, in CAD.

**Fig. 8:**
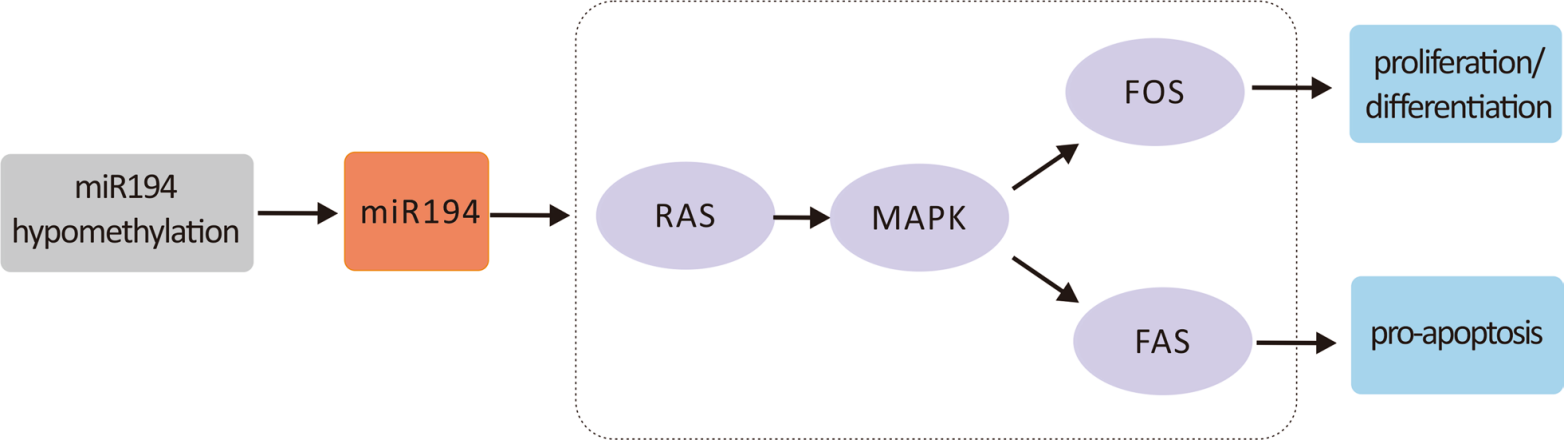
The miR194 promoter-miR194-MAPK signaling pathway

Functional and signal pathway analyses of differential genes revealed that immunity and inflammation-related biological processes played a role in GO enrichment. Using the University of California Santa Cruz (UCSC) database, MiRanda, and gene correlation analyses, we demonstrated that the CAD-related DNA methylation-miRNA-mRNA regulatory network involved 46 DNA methylation sites, 45 miRNAs, and 109 mRNAs. In vitro experiments demonstrated that oxidative damage altered the cell cycle and promoted apoptosis. Interestingly, *miR-194* transfection inhibited apoptosis, restored the cell cycle, and changed the gene expression in the *MAPK* signaling pathway. Demethylation altered *miR194* expression (Fig. 8).

Some studies have investigated methylation in patients with Myocardial infarction (MI). A genomic DNA methylation (DNAm) profile of DNA isolated from whole blood was obtained by analysis with Infinium HumanMethylation450 BeadChip. Three DNAm loci were shown to be significantly correlated between the whole genome and MI [18]. In 2018, the Infinium HumanMethylation450 assay was used to examine the genome-wide DNA methylation profiles in three pairs of samples from patients with acute coronary syndrome and control samples. Methylation-specific polymerase chain reaction (MSP) was also used to validate Sequenom MassARRAY analysis in ACS (acute coronary syndrome), stable coronary artery disease, and control samples. A total of 11,342 differentially methylated CpG sites were identified. Compared with the control sample, the ACS group included 8,865 hypomethylated and 2,477 hypermethylated CpG sites. MSP analysis and Sequenom MassARRAY analysis from 81 ACS samples, 74 stable coronary artery disease samples, and 53 healthy samples confirmed that the reference results of the HumanMethylation450 array significantly corrected the differential CpG methylation of *SMAD3*. These data identified ACS-specific DNA methylation profiles with several novel DM CpG sites, some of which could be candidate markers for early diagnosis of ACS [19]. Other studies have screened and verified the genome in the gene bank and analyzed methylation and related targets to determine the correlation between DNA methylation and gene expression [20–23]. However, DNA methylation changes and validation of ex vivo cells have not been reported in CAD patient screening to validation.

In an in vitro experiment, we showed that *miR-194* transfection inhibited apoptosis, restored the normal cell cycle, and changed gene expression in the *MAPK* signaling pathway. In patients with CAD, the *MAPK* signaling pathway changed accordingly. The inhibition of *miR-194* significantly increased the expression of the related pathways, indicating that *miR-194* may be an important inhibitor of the *MAPK* signaling pathway. *miR-194* is a tumor suppressor similar to *p53* as a typical p53-responsive miRNA. *p53* plays a role in the aging process, and *miR-194* may significantly promote the development of cellular senescence [24]. It has been suggested that *miR-194-5p* is closely related to hypothermia oxygen–glucose deprivation, which is an essential process in ischemic diseases such as CAD [25]. Alongside a middle cerebral artery occlusion Sprague–Dawley rat model, mir-194–1 was downregulated compared with the sham group (0.20 and 0.99) [26]. However, MI downregulates plasma levels of *miR194-5p* in obese and non-obese animals, associated with cardiac function, mitochondrial lipids, and myocardial fibrosis [27]. The expression in MI was 0.60 relative to 1.0 in the control group. In addition, MAPK signal pathway such as p38 and c-jun N-terminal kinase (JNK) which cause inflammation and apoptotic cell death and ERK 1/2 which regulates myocyte differentiation and proliferation, promotes cell survival, and confers tissue protection [28].

The present study has some limitations. First, our sample size for the validation was small. A larger cohort is required to verify the entire network. Although we conducted gene sequencing and validation, all our samples were from the same Hospital. Therefore, it is unclear whether influence would be observed in patients from different areas and races. The validity of the data should be verified in more cohorts. DNA methylation was associated with CAD. We found that a network could connect DNA methylation-miRNA-mRNA. The level of methylation of mRNA could change directly, but not through miRNAs. The detailed mechanism need exploring in future.

In conclusion, we identified differential gene expression profiles of DNA methylation sites, miRNAs, and mRNAs were found in CAD (28,461 methylation sites, 295 miRNAs, and 470 mRNAs). Gene function involved immunity and the inflammatory response pathway. We characterized a specific DNA methylation-miRNA-mRNA regulatory network for CAD and screened the key signaling pathways of the *miR194* promoter-*miR194*-*MAPK* signaling pathway. Through in vitro experiments, we demonstrated the relationship between the *miR194* promoter methylation and *miR194*-*MAPK* regulation. Our findings suggest that the *miR194* promoter and *miR194*-*MAPK* signaling pathway may be related to CAD and may be a potential therapeutic target.

## Materials and methods

### Participant recruitment and sample collection

Our study consisted of 35 patients with CAD and 35 controls from the Guang Anmen Hospital, Beijing, China. The inclusion criteria are as follows: (i) the coronary angiography was to estimate the extent of CAD for all subjects according to the criteria defined by the American Heart Association. At least one major epicardial vessel with > 50% stenosis was defined as CAD, whereas subjects with < 50% stenosis were defined as controls [29, 30] The age is between 45 and 75 y; (ii) exclusion criteria were index event due to uncontrolled hypertension and/or blood pressure remaining ≥ 180/110 mm Hg despite treatment, New York Heart Association class III or IV congestive heart failure irrespective of ejection fraction, or New York Heart Association class II heart failure with left ventricular ejection fraction ≤ 40%, persisting at the end of the run-in period despite treatment, severe valvular heart disease, arrhythmia, cardiomyopathy, and stable angina pectoris, clinically apparent liver, kidney, hematological system, nervous system and mental disease, malignancy within the preceding 3 y, inability to provide informed consent, or comply with study requirements [31].

Risk factors such as smoking history, hypertension, and diabetes: A standardized questionnaire was applied to assess smoking history, hypertension, and diabetes in this study subjects. Smoking history was classified as either “never smoking” or “smoking” (including both former and current smokers). Hypertension was classified as either “non-hypertension” or “hypertension” (including diagnosed as hypertension before and SBP ≥ 140 mmHg and/or DBP ≥ 90 mmHg currently). Diabetes was classified as either “non-diabetes” or “diabetes” (including diagnosed as diabetes before and fasting blood glucose ≥ 7.0 mmol/L or postprandial blood glucose ≥ 11.1 mmol/ L currently) [32]. The severity of CAD was evaluated by Gensini score [33]

The study protocol was approved by the institutional ethics committee of Guang Anmen Hospital, Beijing. The patients volunteered for the research with written consent. All patients signed an informed consent approved by the Institutional Review Board. The patients’ blood samples on an empty stomach were drawn in the morning within 24 h after admission. The blood of the outpatients and the controls were drawn on the morning of the second day after admission. 4 ml venous blood was collected and placed in an EDTA tube. Peripheral blood mononuclear cells (PBNCs) were centrifuged within 6 h.

### DNA and RNA extraction from PBNCs

DNA extraction from PBNCs: 300 μl blood sample was put into a centrifuge tube. We added red blood cell lysate and placed it at room temperature for 5 min. During this period, the mixture was reversed several times and centrifuged. The supernatant was aspirated and discarded. We added 200 μl buffer solution and shook it until completely mixed. 4 μl RNase A solution was added, oscillated for 15 s, and placed at room temperature for 5 min to remove RNA. Then we added 20 μl Proteinase K solution, 200 μl buffer solution GB, 200 μl absolute ethanol. We added the previously mixed liquid into the adsorption column CB3, centrifuged, and poured out the waste liquid. We added 500 μl buffer solution GD to the adsorption column CB3, fully shook for 15 s. Then we added 600 μl rinsing solution PW into the adsorption column CB3, centrifuged, and repeat the operation once. We centrifuged it for 2 min, opened the cover, and put it in the centrifuge tube. Placed it at room temperature for 10 min. Dropped 60 μl ddH_2_O into the middle of the adsorption membrane and place it at room temperature for 5 min. After centrifugation, the solution was added to the adsorption column CB3, placed at room temperature for 2 min, and centrifuged to obtain the extracted DNA solution.

Total RNA extraction from PBNCs: The EDTA tube was inverted and evenly mixed. We placed it in a 15 ml tube and added 3 times the volume of red blood cell lysate, thoroughly mixed it. We placed it at room temperature for 5 min, during which, mixed it several times to ensure complete reaction of reagents. Then we centrifugated it and suction the supernatant. We added red blood cell lysate to 4 ml, thoroughly mixed, and placed it at room temperature for 5 min. After centrifugation, the supernatant was carefully removed, and the white precipitate was retained as PBNCs. 1 ml Trizol was added and mixed with PBNCs, and then transferred to 2 ml centrifuge tube and stored in—80 ℃ refrigerator.

### DNA methylation high-throughput sequencing

We used the Roche Seqcap EPI methylation enrichment kit, following the manufacturer’s instructions [34]. We sequenced the captured bisulfite transformed DNA samples with Illumina hiseq2500. The sequence read lengths were 2 × 150 bp. For data processing, the original data was evaluated by fastqc (FastQC http://www.bioinformatics.bbsrc.ac.uk/projects/fastqc/version0.10.1); clean data were compared to the reference genome using the bsmap software (https://code.google.com/archive/p/bsmap/version2.90). Sam files were sorted according to the chromosomes and loci using the Picard software (http://broadinstitute.github.io/picard/version1.140). Duplicates were marked and removed using the Picard software. The bamtools software (https://github.com/pezmaster31/bamtools/version2.4.0) was used to filter and remove reads that could not be compared. The Bamutil software (https://github.com/statgen/bamUtil/version1.0.14) removed the overlapped area in the middle of the paired reads. Following these steps, we statistically analyzed the optimal comparisons. Based on the methylation detection results of bsmap, the frequency of the methylated C and the unmethylated C at each site was tested using two distribution tests to identify whether the site was a real methylated site. According to the two conditions of sequencing depth ≥ 5 and P ≤ 0.05, the C locus was considered the methylation site. The test results were statistically analyzed. The DMR between the samples was identified using the swDMR software.

### miRNA high-throughput sequencing

We used the NEBNext Small RNA Library Prep Set for Illumina kit (New England Biolabs) to process and build a small RNA library, according to the manufacturer’s instructions [35]. We used the Illumina Hiseq2500 for single-terminal sequencing. We processed data and used FastQC (http://www.bioinformatics.bbsrc.ac.uk/projects/fastqc/version0.10.1) to detect the original sequence. We compared our BLAST results with the RFam database to annotate the miRNA sequence. Clean reads were compared with human precursor/mature miRNA in miRBase (http://www.mirbase.org/version20.0) to screen for known miRNAs. We compared the uncommented sequence with the human genome to analyze its expression distribution. We used Mireap (http://mireap.sourceforge.net/version0.2) to predict potential new miRNAs. The number of sequences of each known miRNA was standardized according to the total number of sequences paired into the miRBase 20.0 database. The number of sequences per million paired sequences (reads per million mapped reads, RPM) was used as the expression amount. FDR correction was applied to p value to obtain Q value as follows: The screening criteria for differential miRNA were as follows: | log2 (fold change) |≥ 1, P value < 0.05.

### mRNA high-throughput sequencing

According to the manufacturer’s instructions, we used the Ribo-Zero Magnetic Gold Kit (Illumina, San Diego, CA, USA) and the NEBNext Ultra RNA Library Prep Kit for Illumina (New England Biolabs) to construct the full transcriptome library. We used the Bioanalyzer 2100 system and qPCR (Kapa Biosystems, Woburn, MA, USA) for quality inspection of the library. We sequenced using the Illumina Hiseq2000/2500 and produced read lengths of 2 × 100/150 bp. The original data was evaluated using fastqc (http://www.bioinformatics.bbsrc.ac.uk/projects/fastqc/version0.10.1), and compared with the reference genome by the TopHat 2.0 program (http://tophat.cbcb.umd.edu/version2.0.10). After the transcripts of each sample were assembled separately, the transcripts of all samples were summarized and merged using the command of cuffmerge. Then, the ensembl transcript database was used as the annotation reference for mRNA, and the number of sequences in each transcript was standardized according to the total length and samples. The number of sequences in every 1 million pairs to every 1000 bases in exon was used as the expression amount. FDR correction was applied to p value to obtain Q value as follows: The screening criteria for differential mRNA were as follows: P value < 0.05 and Q value ≤ 0.05.

### Construction of a regulatory network among genes

After obtaining the differential expression of DNA methylation, miRNA, and mRNA among the groups according to the regulatory relationships between genes, the gene regulatory network was screened using the UCSC database (https://genome.ucsc.edu/) and the MiRanda miRNA target gene database (http://www.microrna.org/microrna/version3.3a). Then, we chose miRNAs as follows: | log2 (fold change) |≥ 1, P value < 0.05 and Q value ≤ 0.05. The criteria for differential mRNA were as follows: P value < 0.05 and Q value ≤ 0.05.

### Verification of the methylation level of related sites using pyrosequencing

The reaction system was set up for bisulfite transformation: the transformation was carried out on a 9700 type PCR instrument (with a hot cover), and the DNA was frozen and preserved after treatment. We used the pyromark PCR kit as follows: First, the single strain PCR products were purified, and the primers were annealed. PCR products were retained for pyrosequencing on the pyromark ID instrument. The assay and run were established in the pyromark CpG software. We prepared the related items and sequences. Sequencing results were analyzed.

### Detection of the methylation level of *miR-194* and miR-200 promoter using sequencing

The genomic DNA of tissue or peripheral blood was extracted using the phenol–chloroform method. The DNA concentration, OD 260, OD 280, and OD 260/280 were measured using a Nanodrop 1000 spectrophotometer. The DNA sample OD 260/280 used in this experiment was between 1.8–1.9, regarded as qualified DNA. The DNA samples were treated with bisulfite, amplified by PCR, and reacted with SAP. The 384 pore plates were placed into the sample adding instrument. After the parameter setting is completed, the sample was spotted on the chip. We then entered information about the target sequence in the epicity software. After sampling, the chip was placed in the mass spectrometer, and methylation analysis was performed according to the molecular weight difference of C and T bases in the fragment. The methylation ratio of MS was obtained from Epityper software version 1.0 (https://www.cd-genomics.com/EpiTYPER-DNA-Methylation-Analysis.html Sequenom, San Diego, CA) (Additional files 1, 2).

### qRT-PCR

The cells were collected and treated 72 h later. Total RNA was extracted by the Trizol method and then reverse transcribed into cDNA for PCR. PCR reaction system: cDNA (diluted 10 times) 4 μL, SYBR Green 5 μL, primer 0.2 μL + primer (10 μmol/L), 0.2 μL each, water 0.6. μL, total 10 μL of PCR conditions: 95 °C for 5 min; 95 °C for 10 s, 60 °C for 20 s, a total of 50 cycles; 5 °C for 10 s, 60 °C for 10 s, and 40 °C for 30 s. The CT values of the target and control genes were automatically collected by the quantitative fluorescence analyzer, and the relative mRNA expression was calculated using the 2^−ΔΔCT^.

### Cell culture and grouping

HUVECs were cultured in DMEM containing 10% fetal bovine serum, 100 U/mL penicillin, and 100 mg/L streptomycin. The medium was cultured in a 5% CO_2_ incubator at 37 °C and changed daily. The third to eighth-generation cells were used in all experiments. HUVECs grew to about 80% fusion degree. After centrifugation, a 1.5 mL DMEM medium (4 °C precooling) containing 10% DMSO was added to the cell precipitate. After blowing and mixing, the cell suspension was transferred to a 2 mL cryopreservation tube and stored at—80 °C.

HUVECs were divided into four groups: blank, H_2_O_2_ model, 5-aza, and transfection groups. The H_2_O_2_ model group was treated with 100 μL of H_2_O_2_ for 3 h. The 5-aza group was treated with 50 μM 5-aza for 24 h. Cell samples were collected 24 h after treatment with H_2_O_2_ for 3 h.

### Liposome transfection

The cells were transfected in an 80% fusion state. The Lipofectamine RNAiMAX liposomes were mixed in the proportion of 6 μL:100 μL in the Opti-MEM solution; the siRNA was mixed in the proportion of 2 μL:100 μL in the Opti-MEM solution; the siRNA was mixed in the proportion of 1:1 in the Lipofectamine RNAiMAX liposome mixture. The mixture was stored at room temperature for 5 min, added to the cells, placed in the incubator for 48 h, and collected.

### Annexin V/PI determination of apoptosis

The HUVECs were divided into groups of 11. Forty-eight hours after infection with the lentiviral vector control and lentiviral-Bmi1-shRNA and 24 h after treatment with cisplatin, cells were centrifuged for 5 min. We added 100 μL of binding buffer to cells (1 × 10^6^) in 1.5 mL centrifuge tubes. Annexin V was incubated at 5 μL, following which, we added 3 μL PI. Apoptosis was analyzed using flow cytometry with 400 μL binding buffer.

### Statistical analyses

We used SPSS19.0 for all statistical analyses. Data were expressed as mean ± standard deviation. We used independent sample t-tests to analyze the normal distribution between groups. We used the rank-sum test to analyze the non-normal distribution. For multiple comparisons, the quantitative data of normal distributions were analyzed by variance analysis and the Q test. The quantitative data of non-normal distribution were analyzed by nonparametric tests.

## Supplementary Information

**Additional file 1**. 171 DE miRNAs were downregulated.

**Additional file 2**. 124 DE miRNAs were upregulated.

## Data Availability

The datasets analyzed during the current study are available in the the National Library of Medicine repository, https://submit.ncbi.nlm.nih.gov/subs/sra/SUB8748589/overview.

## Abbreviations

ACS: Acute coronary syndrome
CAD: Coronary artery disease
CpG: Cytosine-phosphate-guanine
DBP: Diastolic blood pressure
DEGs: Differentially expressed genes
DMRs: Differentially methylated regions
DNAm: DNA methylation
DNMT: DNA methyltransferase
FBG: Fasting blood glucose
FH: Familial hypercholesterolemia
GO: Gene ontology
HDL-C: High-density lipoprotein cholesterol
HUVEC: Human Umbilical Vein Endothelial Cell
KEGG: Kyoto Encyclopedia of Genes and Genomes
LDL-C: Low-density lipoprotein cholesterol
MAPK: Mitogen-activated protein kinase
miRNA: MicroRNA
MI: Myocardial infarction
MSP: Methylation-specific polymerase chain reaction
PBNCs: Peripheral blood mononuclear cells
PCA: Principal components analysis
PI: Propidium iodide
qRT-PCR: Quantitative real-time polymerase chain reaction
SBP: Systolic blood pressure
T2DM: Type 2 diabetes mellitus
TC: Total cholesterol
TCM: Traditional Chinese Medicine
TG: Triglyceride
UCSC: The University of California Santa Cruz
